# Reprogramming to Pluripotency through a Somatic Stem Cell Intermediate

**DOI:** 10.1371/journal.pone.0085138

**Published:** 2013-12-27

**Authors:** Adele G. Marthaler, Ulf Tiemann, Marcos J. Araúzo-Bravo, Guangming Wu, Holm Zaehres, Jung Keun Hyun, Dong Wook Han, Hans R. Schöler, Natalia Tapia

**Affiliations:** 1 Department of Cell and Developmental Biology, Max Planck Institute for Molecular Biomedicine, Münster, Germany; 2 Department of Nanobiomedical Science, Dankook University Graduate School, Cheonan, Republic of Korea; 3 Department of Rehabilitation Medicine, Dandook University, Cheonan, Republic of Korea; 4 Department of Stem Cell Biology, School of Medicine, Konkuk University, Seoul, Republic of Korea; 5 Medical Faculty, University of Münster, Münster, Germany; University of Kentucky, United States of America

## Abstract

Transcription factor-based reprogramming can lead to the successful switching of cell fates. We have recently reported that mouse embryonic fibroblasts (MEFs) can be directly reprogrammed into induced neural stem cells (iNSCs) after the forced expression of *Brn4*, *Sox2*, *Klf4*, and *Myc*. Here, we tested whether iNSCs could be further reprogrammed into induced pluripotent stem cells (iPSCs). The two factors *Oct4* and *Klf4* were sufficient to induce pluripotency in iNSCs. Immunocytochemistry and gene expression analysis showed that iNSC-derived iPSCs (iNdiPSCs) are similar to embryonic stem cells at the molecular level. In addition, iNdiPSCs could differentiate into cells of all three germ layers, both *in vitro* and *in vivo*, proving that iNdiPSCs are *bona fide* pluripotent cells. Furthermore, analysis of the global gene expression profile showed that iNdiPSCs, in contrast to iNSCs, do not retain any MEF transcriptional memory even at early passages after reprogramming. Overall, our results demonstrate that iNSCs can be reprogrammed to pluripotency and suggest that cell fate can be redirected numerous times. Importantly, our findings indicate that the induced pluripotent cell state may erase the donor-cell type epigenetic memory more efficiently than other induced somatic cell fates.

## Introduction

Mouse embryonic fibroblasts (MEFs) can be reprogrammed into induced pluripotent stem cells (iPSCs) following the overexpression of the four transcription factors *Oct4*, *Sox2*, *Klf4*, and *Myc* [1]. The same combination of transcription factors under different culture conditions was used to convert MEFs into induced epiblast stem cells (iEpiSCs) [2]. Furthermore, neural stem cells (NSCs), which exhibit high levels of endogenous *Sox2* expression, have been reprogrammed into iPSCs by using only *Oct4* and *Klf4* [3]. In addition to reprogramming to pluripotency, different combinations of transcription factors have been shown to directly switch somatic cell fates in the absence of an intermediate pluripotent cell state. Indeed, fibroblasts have been directly converted into different types of post-mitotic somatic cells such as neurons and cardiomyocytes [4,5]. In addition, we have recently reported the direct reprogramming of MEFs into induced neural stem cells (iNSCs) that can self-renew [6]. Interestingly, reprogramming of induced somatic cells into iPSCs has yet to be described.

At early time points after reprogramming, iPSCs maintain a donor-cell type epigenetic memory that can potentially bias their differentiation potential toward the cell lineage of origin [7-9]. Furthermore, contradictory results have been reported on whether it is possible to erase this iPSC somatic memory with further passaging [7,10]. Remarkably, the epigenetic memory retained in somatic cell types generated through direct reprogramming has not been studied extensively. In our previous report, we showed that two fibroblast marker genes were expressed in iNSCs at early, but not at late, passages after conversion [6]. However, the impact of the remaining MEF transcriptional signature on iNSC functionality was not evaluated. In fact, an analysis comparing the residual donor epigenetic memory of the same cell types generated by either direct reprogramming or iPSC differentiation has never been performed.

In the current study, we first converted MEFs into iNSCs and then reprogrammed these iNSCs into iPSCs (iNdiPSCs), demonstrating that somatic cell types generated by a direct reprogramming approach can be further reprogrammed to pluripotency. In contrast to iNSCs, iNdiPSCs did not exhibit any residual MEF transcriptional memory at early passages, suggesting that the pluripotent cell state can reset the somatic transcriptional network more efficiently than the induced somatic stem cell state.

## Results

### iNSC-derived iPSCs (iNdiPSCs) Exhibit Pluripotency *In Vitro* and *In Vivo*

**Figure 1:**
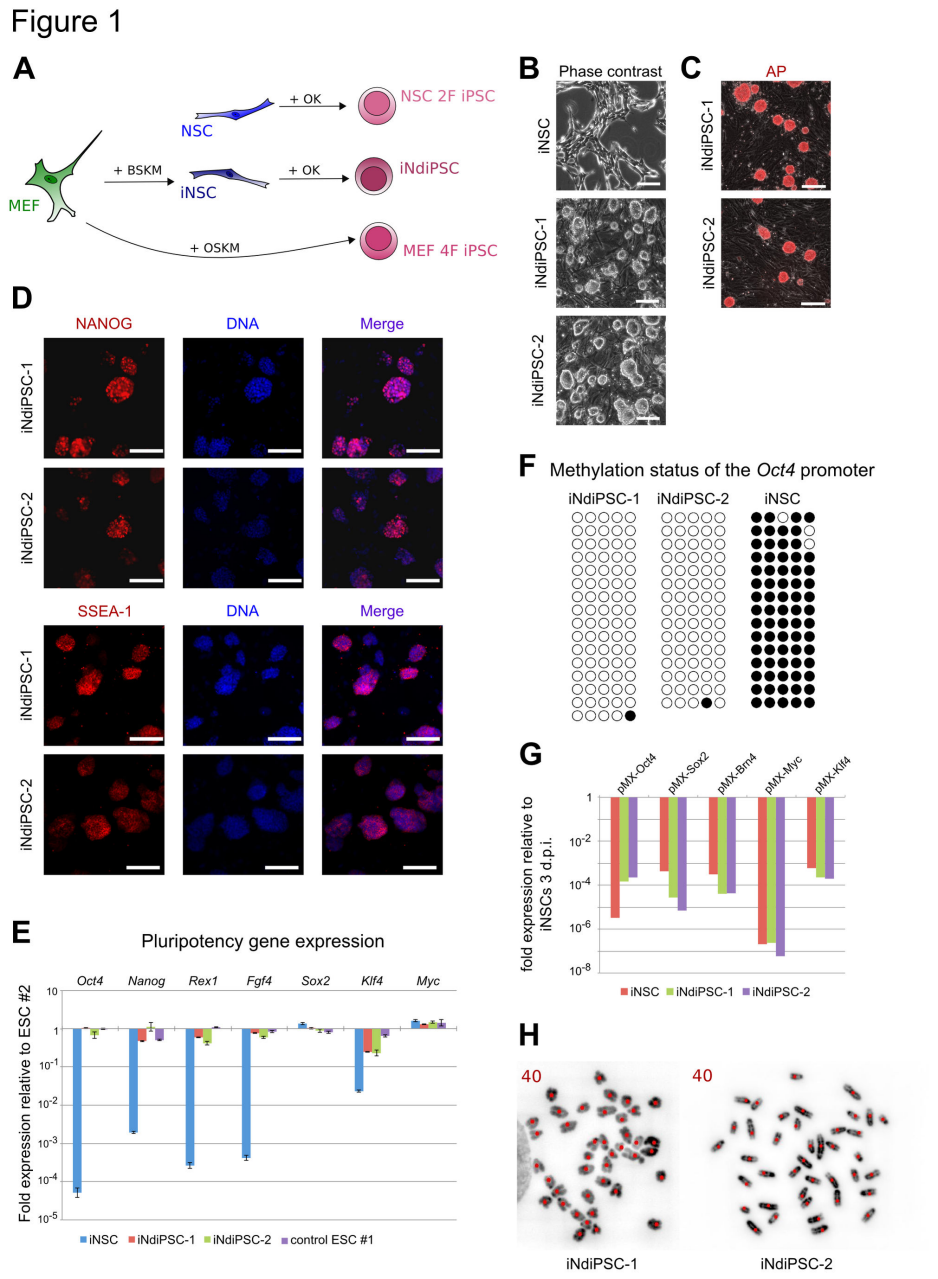
Generation and characterization of iNdiPSCs. (**A**) Scheme of the experimental approach. MEFs can be directly converted into iNSCs after the forced expression of Brn4 (B), Sox2 (S), Klf4 (K), and Myc (M). The overexpression of Oct4 (O) and Klf4 leads to the reprogramming of iNSCs and brain-derived NSCs into iNdiPSCs and NSC 2F iPSCs, respectively. Finally, MEFs can be reprogrammed into iPSCs using Oct4, Sox2, *Klf4*, and Myc. (**B**) Phase-contrast images of iNSCs and iNdiPSCs. Scale bars, 200 µm. (**C**) Alkaline phosphatase staining of iNdiPSC clones 1 and 2. Scale bars, 200 µm. (**D**) Immunofluorescence microscopy images of iNdiPSC-1 and -2 using antibodies directed against NANOG and SSEA-1. Scale bars, 150 µm. (**E**) Expression levels of pluripotency genes measured by qRT-PCR. Data were normalized to *Rpl37a* and plotted relative to control ESCs #2. Error bars indicate standard deviation of triplicate values. (**F**) Methylation analysis of the Oct4 promoter in iNdiPSC-1 and -2 and iNSCs by bisulfite sequencing PCR. Open and filled circles represent unmethylated and methylated CpGs, respectively. (**G**) Silencing of exogenous transgenes shown by qRT-PCR. Data were normalized to *Rpl37a* and plotted relative to iNSCs three days post-infection (3 dpi). (**H**) Chromosome spread of iNdiPSC-1 and -2 showing a stable karyotype of 40 chromosomes. Red dots indicate counted chromosomes.

**Figure 2:**
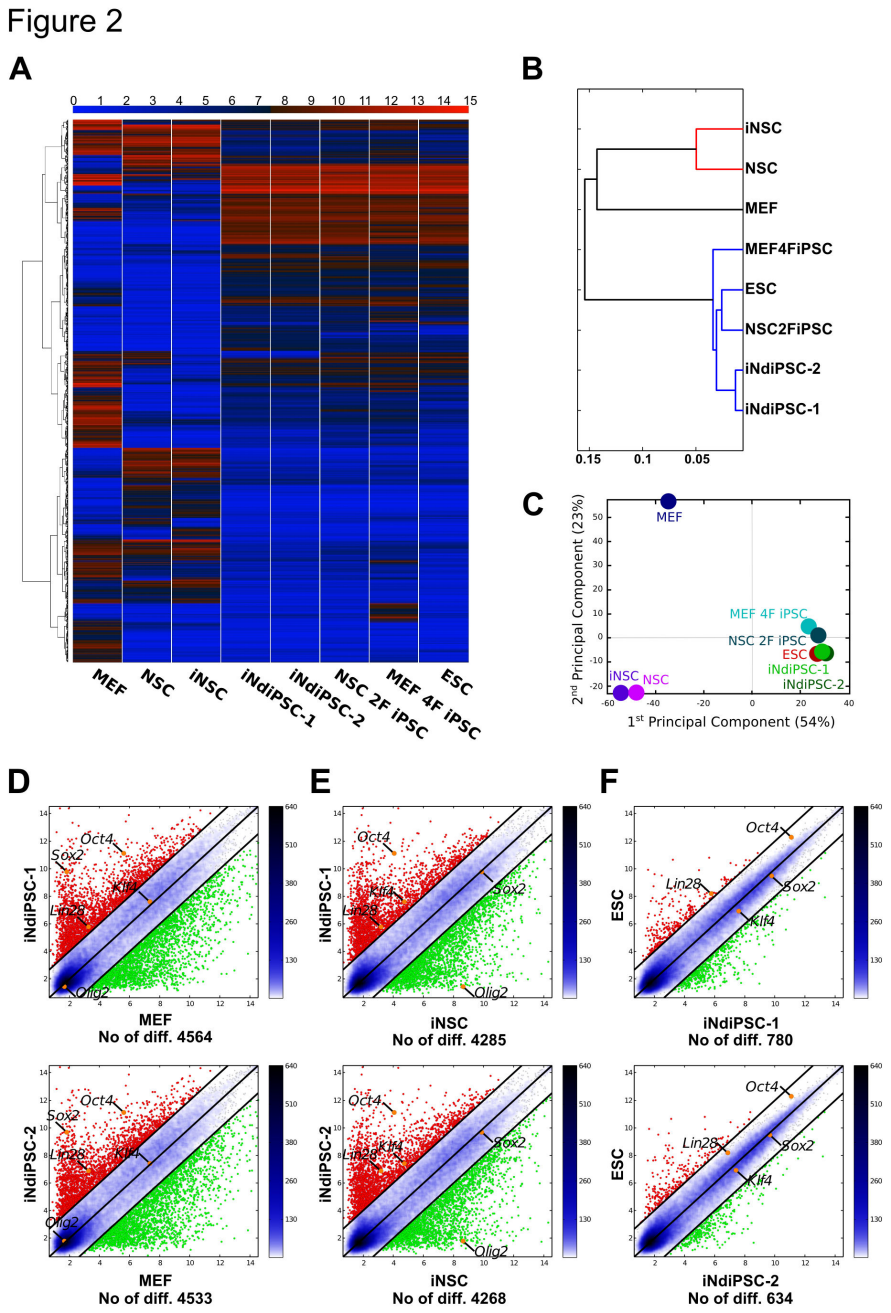
iNdiPSCs exhibit a pluripotent global gene expression profile. (**A**) Heat map of microarray data showing similar gene expression patterns of iNdiPSC-1 and -2 (both passage 10) compared with ESCs, NSC 2F iPSCs (passage 7, 2F: Oct4 and Klf4), and MEF 4F iPSCs (passage 7, 4F: Oct4, Sox2, *Klf4*, and Myc). MEFs, NSCs, and iNSCs serve as controls. Color bar at the top indicates gene expression in log_2_ scale. Red and blue colors represent high and low expression levels, respectively. (**B**) Hierarchical clustering and (**C**) principal component analysis (PCA) of the different cell types depicted in (**A**). (**D**) – (**F**) Pairwise scatter plots comparing the gene expression profile of iNdiPSCs-1 and -2 with MEFs (**D**), iNSCs (**E**), and ESCs (**F**). Specifically highlighted are the pluripotency marker genes Oct4, Sox2, *Lin28*, and Klf4. Black lines indicate boundaries of 2-fold difference in gene expression levels. Scattering density is indicated in the bar on the right: the higher the density, the darker the color. Gene expression levels are depicted in log_2_ scale. The number of differentially expressed genes (No of diff.) is listed below each plot.

**Figure 3:**
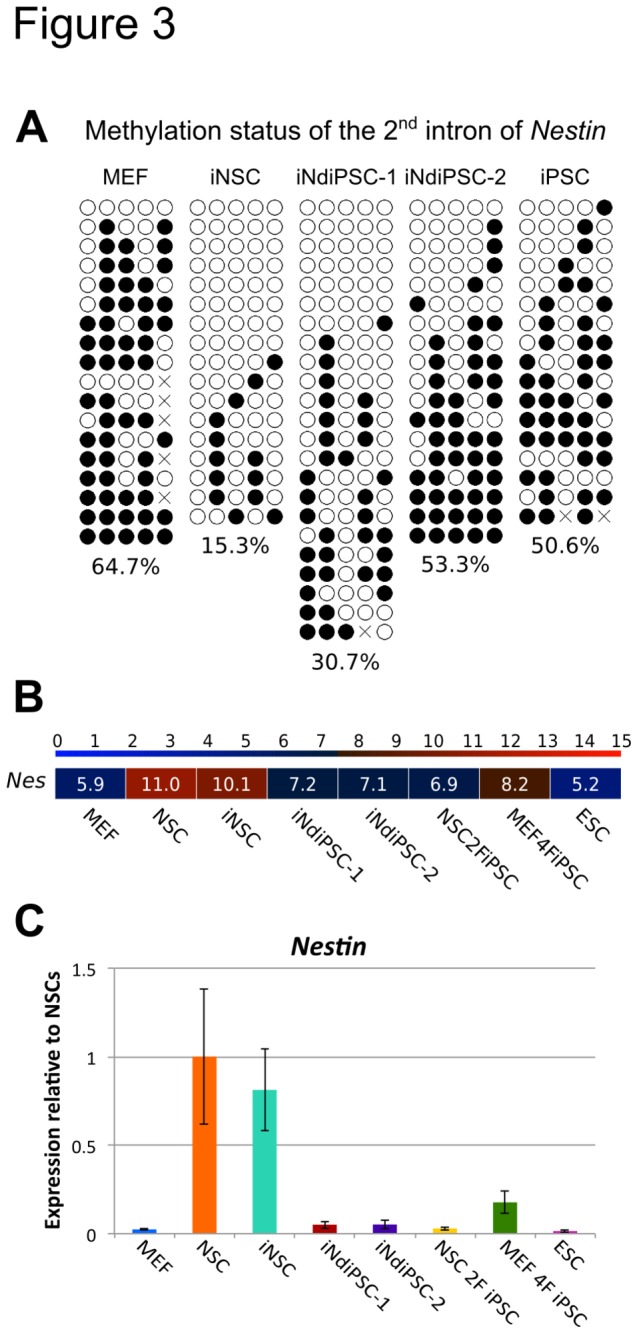
Promoter and transcription analysis of Nestin in iNdiPSCs vs. **iNSCs**. (**A**) Methylation analysis of the second intron of Nestin in MEFs, iNSCs, iNdiPSC-1 and -2, and control iPSCs. Open and filled circles represent unmethylated and methylated CpGs, respectively. X represents an undetermined CpG. The percentage of the methylation is depicted beneath each sample. (**B**) Heat map of Nestin expression according to microarray data. Color bar at the top indicates gene expression in log_2_ scale. Red and blue colors represent high and low expression levels, respectively. (**C**) Expression levels of Nestin measured by qRT-PCR. Data were plotted relative to NSCs. Error bars indicate standard error of two different housekeeping genes (*Gapdh* and *Actb*).

**Figure 4:**
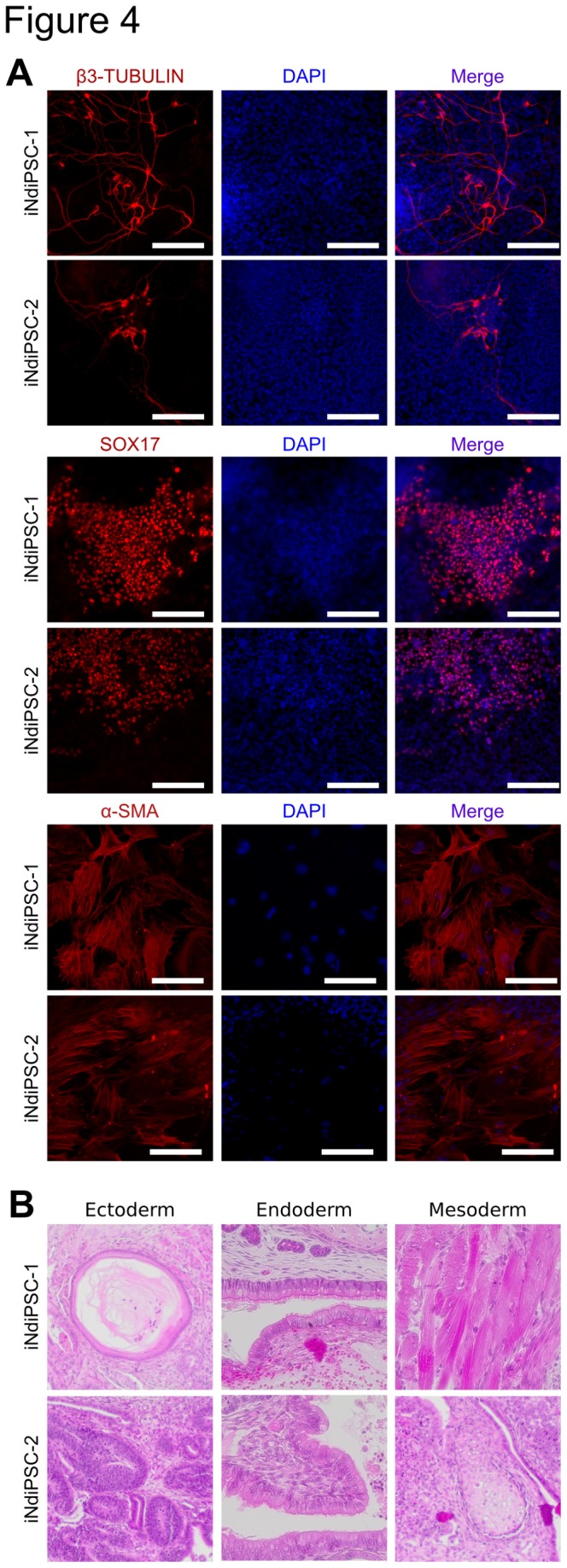
iNdiPSCs can differentiate into cells of all three germ layers *in* *vitro* and *in* *vivo*. (**A**) Immunofluorescence microscopy images of iNdiPSC-1 and -2-differentiated embryoid bodies using antibodies directed against TUBB3/β3-TUBULIN (ectoderm), SOX17 (endoderm), and ACTA2 (α-SMA) (mesoderm). Scale bars, 150 µm. (**B**) Teratoma analysis of iNdiPSC-1 and -2 showing tissues of all three germ layers: ectoderm (neural rosette and keratinocytes), endoderm (gut-like endothelium), and mesoderm (muscle and cartilage).

We have recently reported the direct conversion of MEFs into iNSCs by using four transcription factors [6]. As brain-derived NSCs can be readily reprogrammed into iPSCs by using the two factors *Oct4* and *Klf4* [3], we sought to assess whether iNSCs could be further reprogrammed into iPSCs. To this end, iNSCs were transduced with replication-defective retroviral particles coding for only *Oct4* and *Klf4* (Figure 1A). We observed the first iPSC colonies 13 days after transduction and termed them iNSC-derived iPSCs (iNdiPSCs). Two independent experiments were performed and the overall reprogramming efficiency was found to range from 0.05% to 0.088% (Table S1). As expected, no iNdiPSC colonies were detected in the non-transduced control wells. Two independent clones (iNdiPSC-1 and -2) were picked, expanded (Figure 1B), and further characterized. iNdiPSC-1 and -2 stained positive for the pluripotency markers alkaline phosphatase (Figure 1C), NANOG, and SSEA-1 (Figure 1D). Furthermore, the expression levels of several pluripotency markers (*Oct4*, *Nanog*, *Rex1*, *Fgf4*, *Sox2*, *Klf4*, and *Myc*) in both iNdiPSC clones were similar to the levels observed in two independent control ESC lines (Figure 1E). In contrast to the starting iNSC population, the *Oct4* promoter was fully demethylated in the iNdiPSC clones (Figure 1F). As silencing of retroviral vectors is a hallmark of pluripotent stem cells [1], we examined transgene expression in both iNdiPSC clones and found the retroviral transgenes coding for *Klf4* and *Oct4* to be effectively silenced (Figure 1G). Moreover, we confirmed that the transgenes *Sox2, Brn4* and *Myc*, which were used for iNSC induction, were silenced in the iNSC population and were not reactivated upon iNdiPSC formation (Figure 1G). In addition, the iNdiPSCs exhibited a normal karyotype of 40 chromosomes (Figure 1H). Microarray analysis was then performed to compare the iNdiPSCs global gene expression profile in several pluripotent cell lines such as ESCs, MEF 4F iPSCs (MEF-derived iPSCs by using *Oct4*, *Sox2*, *Klf4*, and *Myc*), and NSC 2F iPSCs (NSC-derived iPSCs by using *Oct4* and *Klf4*) (Figure 1A). iNdiPSCs displayed a transcriptomic profile that clustered with the pluripotent cell lines, but not the parental iNSCs, as shown in the heat map, hierarchical unsupervised clustering, and principal component analysis (Figure 2A-C). Scatter plot pairwise comparisons of iNdiPSCs with ESCs, MEFs, or iNSCs provided further confirmation that iNdiPSCs were successfully reprogrammed to pluripotency. Indeed, iNdiPSCs differ greatly from iNSCs and the starting MEF population, but are very similar to control ESCs (Figure 2D-F). To demonstrate the loss of epigenetic marks in the reprogrammed iNdiPSCs, we analyzed the methylation status of the second intron of *Nestin*, which has been shown to be completely unmethylated in NSCs and iNSCs [11]. The second intron of *Nestin* was methylated in iNdiPSCs to levels similar to those of control iPSCs and MEFs (Figure 3A). The methylation status correlated with *Nestin* gene expression as assessed by microarray analysis (Figure 3B) and confirmed by qRT-PCR (Figure 3C). Finally, we investigated whether iNdiPSCs could differentiate *in vitro* and *in vivo* into derivatives of all three germ layers. The iNdiPSC *in vitro* differentiation potential was tested by using embryoid body formation. Immunocytochemistry revealed that both iNdiPSC clones could differentiate into ectoderm (β3-TUBULIN), endoderm (SOX17), and mesoderm (α-SMA) (Figure 4A). To assess their differentiation potential *in vivo*, iNdiPSCs were subcutaneously injected into SCID mice. After 4 weeks, teratomas containing tissues representative of all three germ layers had formed (Figure 4B). Taken together, cellular, molecular, and differentiation analyses proved that iNdiPSCs are *bona fide* pluripotent cells.

### iNdiPSCs do not Display MEF or iNSC Transcriptional Memory

**Figure 5:**
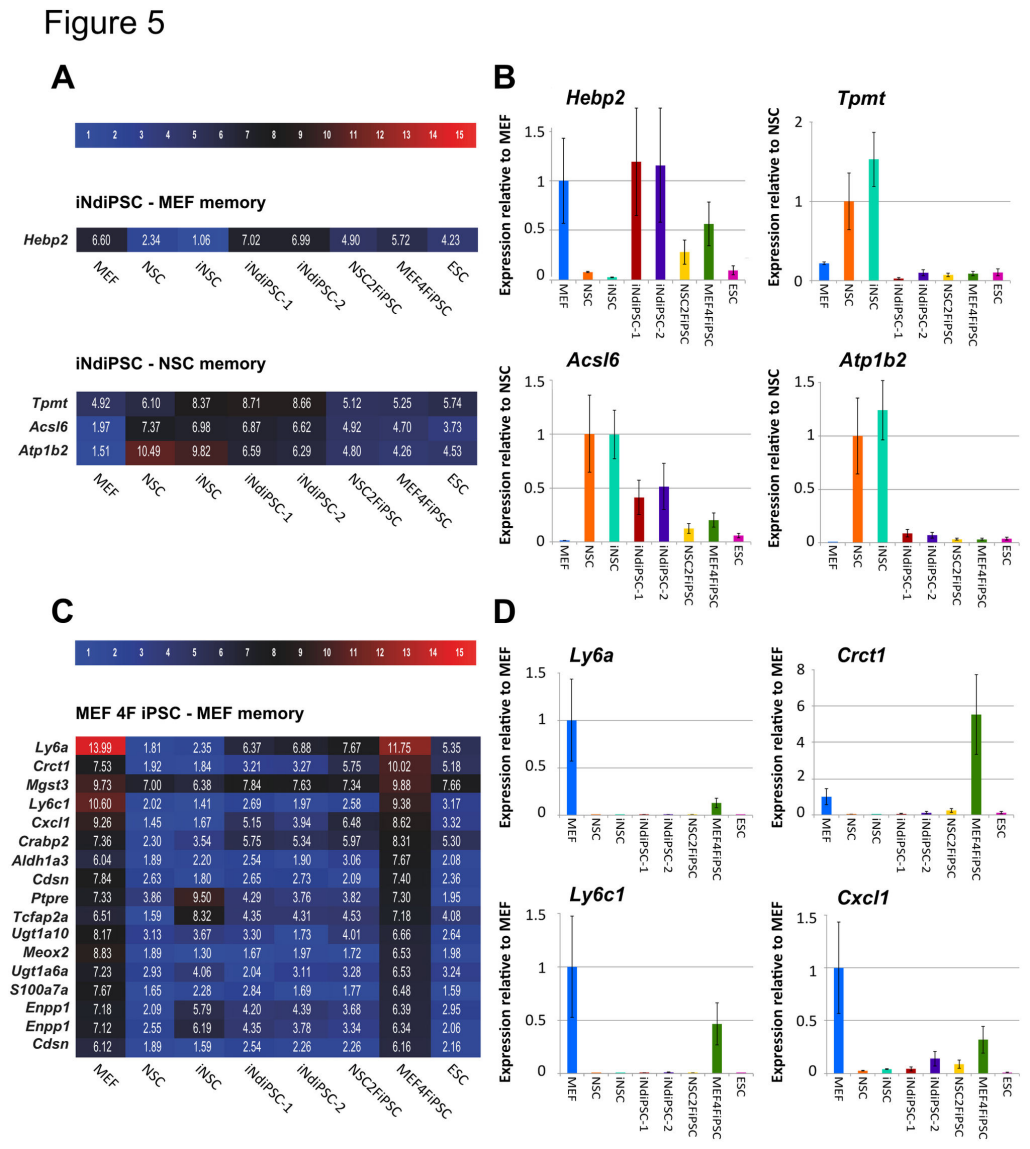
iNdiPSCs do not retain a donor-cell type transcriptional memory. (**A**) Heat map of potential memory genes in iNdiPSCs. The filter criteria are listed in the Methods section. Color bar at the top indicates gene expression in log_2_ scale. Red and blue colors represent high and low expression levels, respectively. (**B**) Gene expression of potential NSC or MEF memory genes in iNdiPSCs was verified by qRT-PCR. Data were plotted relative to NSCs or MEFs in the case of NSC or MEF memory, respectively. Error bars indicate standard error of two different housekeeping genes (*Gapdh* and *Actb*). (**C**) Heat map of potential MEF memory genes in MEF 4F iPSCs. The filter criteria are listed in the Methods section. Color bar at the top indicates gene expression in log_2_ scale. Red and blue colors represent high and low expression levels, respectively. (**D**) Gene expression of selected potential MEF memory genes in MEF 4F iPSCs was verified by qRT-PCR. Data were plotted relative to MEFs. Error bars indicate standard error of two different housekeeping genes (*Gapdh* and *Actb*).

In our previous study, we showed that iNSCs exhibited residual MEF transcriptional memory at early stages after reprogramming [6]. To confirm this finding, we screened the global gene expression data, shown in Figure 2, for genes expressed in MEFs and iNSCs, but not in NSCs or ESCs. The filter criteria are thoroughly explained in the Methods section. Indeed, early-passage iNSCs expressed 61 MEF-specific genes (Figure S1), from which the expression of six genes (*Hoxc9*, *Hoxc6*, *Adcy2*, *Cryab*, *Pdzrn3*, and *Anxa5*) was confirmed by qRT-PCR (Figure S2). Consequently, we investigated whether early-passage iNdiPSCs still maintain the MEF transcriptional memory that we detected in the iNSC population. Therefore, we screened for genes expressed in MEFs and iNdiPSCs, but not in NSCs or ESCs. Only the gene *Hebp2* fulfilled the filter criteria and its expression was confirmed by qRT-PCR (Figure 5A and B). This gene, however, was not expressed in the iNSC intermediate cell type, suggesting that *Hebp2* was nonspecifically upregulated during iPSC derivation. Consequently, we concluded that iNdiPSCs do not exhibit any MEF-transcriptional memory and that the MEF memory retained in early-passage iNSCs is lost when the cells are further reprogrammed into a pluripotent cell type. In addition, we assessed whether early-passage iNdiPSCs retain the iNSC memory of the iNSC intermediate. Hence, we sorted for genes expressed in iNSCs and iNdiPSCs, but not in MEFs or ESCs. Only three potential genes were found, namely *Tpmt, Acsl6*, and *Atp1b2* (Figure 5A). However, qRT-PCR analysis did not confirm the expression pattern of *Tpmt* in iNdiPSCs (Figure 5B). In addition, the expression levels of *Atp1b2* were comparable in all pluripotent cell lines, excluding a NSC-specific memory (Figure 5B). Moreover, residual *Acsl6* expression was detected not only in iNdiPSCs, but also in MEF 4F iPSCs (Figure 5B). As MEFs do not express *Acsl6*, we speculated that expression of *Acsl6* might be a nonspecific effect of the pluripotency induction process rather than a residual NSC-specific transcriptional signature. Thus, we concluded that iNdiPSCs do not exhibit any MEF or iNSC transcriptional memory. As a control, we assessed the residual expression of MEF- and NSC-specific genes in MEF 4F iPSCs and NSC 2F iPSC, respectively. As shown in Figure 5C, MEF 4F iPSCs express 23 MEF-specific genes, and the expression of four of these genes (*Ly6a*, *Crct1*, *Ly6c1*, and *Cxcl1*) was validated by qRT-PCR (Figure 5D). In the case of NSC 2F iPSCs, no genes fulfilled the filter criteria for NSC transcriptional memory. Thus, our results suggest that early-passage iNSCs retain a stronger cell-of-origin transcriptional memory than early-passage iPSC lines.

### iNdiPSCs do not Present any Bias in their Differentiation Potential

**Figure 6:**
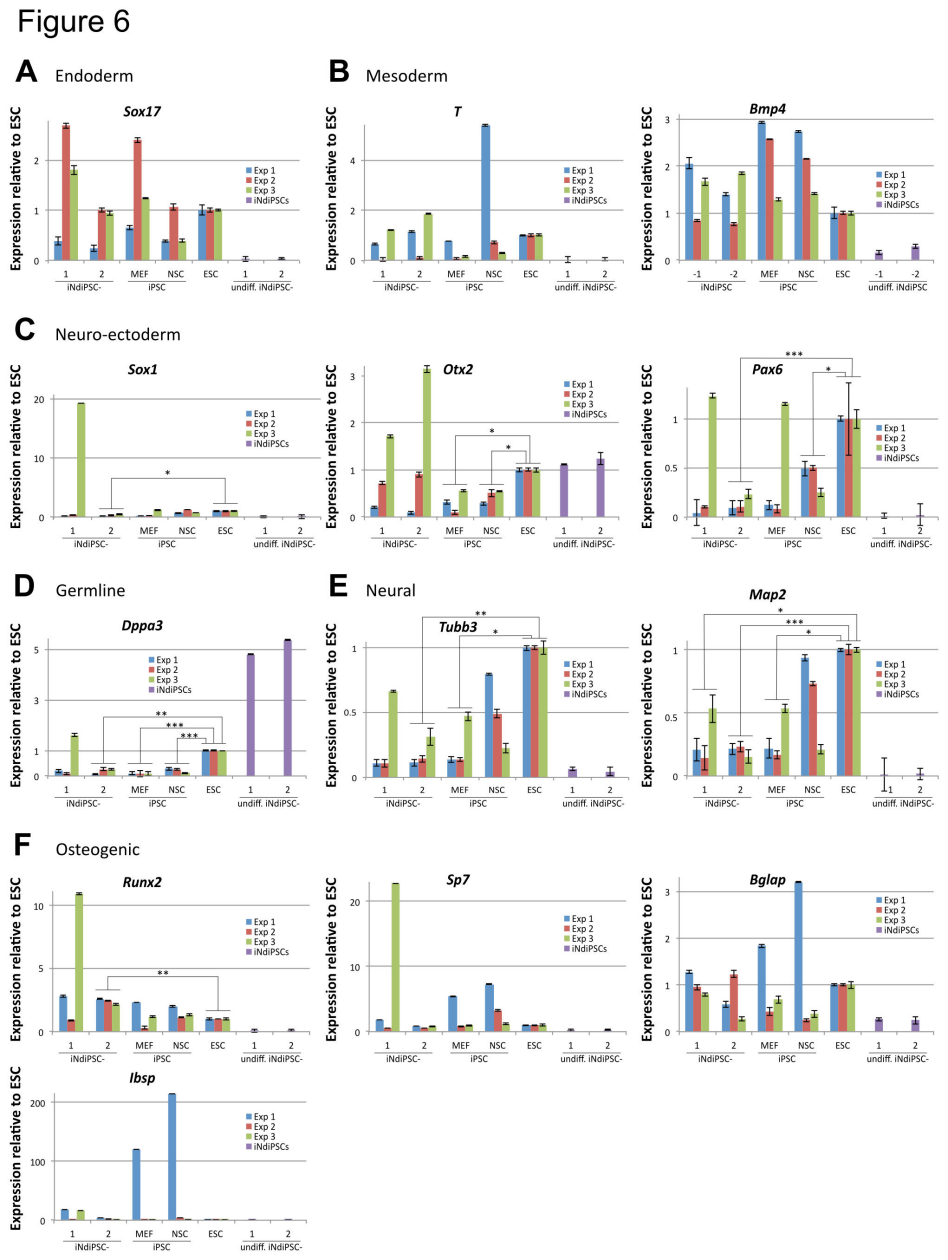
iNdiPSCs do not show a differentiation bias. Differentiated embryoid bodies of iNdiPSC-1 and -2, MEF 4F iPSCs (MEF iPSC), NSC 2F iPSCs (NSC iPSC) and control ESCs (ESC) were analyzed for the expression of (**A**) endoderm, (**B**) mesoderm, (**C**) (neuro) ectoderm-, (**D**) germline, (**E**) neural, and (**F**) osteogenic markers. The data of three independent experiments (Exp 1, Exp 2, and Exp 3) were plotted relative to ESCs. Additionally undifferentiated iNdiPSCs (undiff. iNdiPSC-1 and -2) are depicted as a control. Error bars indicate standard error of triplicate values. All data were normalized to RpI37a. Statistical significance was determined by performing Student’s *t*-test and indicated with asterisks *p ≤ .01, **p ≤ .001, ***p ≤ .0001.

Even in the absence of a detectable transcriptional memory, iPSCs can retain donor-cell type epigenetic marks that predispose for differentiation toward the cell lineage of origin [8,10]. Therefore, we investigated whether iNdiPSCs exhibit a differentiation potential bias. iNdiPSCs, MEF 4F iPSCs, NSC 2F iPSCs, and ESCs were differentiated *in vitro* by using embryoid body formation, a method that promotes stochastic differentiation into all germ layers. Three independent experiments were conducted to analyze statistically significant variations. At two weeks of differentiation, the expression of endoderm (*Sox17*), mesoderm (*T* and *Bmp4*), neuro-ectoderm (*Sox1*, *Otx2*, and *Pax6*), germline (*Dppa3*), neural (*Tubb3* and *Mpa2*), and osteogenic (*Runx2*, *Sp7, Bglap*, and *Ibsp*) marker genes was analyzed by qRT-PCR (Figure 6A-F). Undifferentiated iNdiPSCs that do not express the selected lineage-specific marker genes were used as a negative control. Of note, *Dppa3* and *Otx2* are also present in pluripotent cells [12,13] and could, therefore, be detected in undifferentiated iNdiPSCs. In comparison with ESCs, iNdiPSCs did not show a significant increase in any of the studied genes, thus excluding a preferential differentiation bias into any specific cell lineage. As an exception, *Runx2* expression levels were significantly higher in iNdiPSC clone 2, but not in clone 1. However, iNdiPSC clone 2 did not present a predisposition toward the osteogenic lineage since the other osteogenic markers were not upregulated. Furthermore, MEF 4F iPSCs, NSC 2F iPSCs, and iNdiPSC clone 2 exhibited a significant decrease in the expression levels of several neuro-ectodermal, neural and germline markers (Figure 6C, E and D). In contrast, the differentiation profile of iNdiPSC clone 1 resembled control ESCs, demonstrating that iNdiPSCs are not intrinsically impaired in the differentiation toward the neural lineage. Finally, no significant differences between either iNdiPSC clone and MEF 4F iPSCs or NSC 2F iPSCs could be determined in all analyzed marker genes. Therefore, the differentiation potential of iNdiPSCs is similar to that of control iPSCs derived from MEFs and NSCs. In summary, reprogramming to pluripotency resets the MEF, NSC, and iNSC transcriptional program to an equivalent state and eliminates any bias in the differentiation potential.

## Discussion

Primed pluripotent iEpiSCs, which are converted directly from MEFs, have been further reprogrammed into naïve pluripotent iPSCs after the forced expression of *Klf4* [2]. However, the ability of induced somatic cells to be subsequently reprogrammed into the pluripotent cell state has not been previously tested. Our study shows that fibroblast-derived iNSCs can be further reprogrammed to pluripotency using two transcription factors, as described for brain-derived NSCs [3]. Therefore, our results demonstrate that several cell fates can be consecutively induced from the same initial cell type.

Several studies have reported that iPSCs retain the transcriptional and epigenetic memory of the cell of origin and that this residual memory can influence their differentiation potential [10]. Our analysis indicates that iPSCs derived from MEFs, NSCs, and iNSCs do not exhibit the transcriptional signature of the initial cell type. Interestingly, some MEF-specific genes such as *Crtc1* in MEF 4F iPSCs or *Hoxc9*, *Adcy2*, *Pdzrn3*, and *Hoxc6* in iNSCs were expressed at higher levels in MEF 4F iPSCs and iNSCs compared with the initial MEF population. This gene expression pattern does not correlate with transcriptional memory, for which gene expression levels should be similar or lower than those of the starting cell type. Rather, we propose that this upregulation in expression levels might be a nonspecific effect of the reprogramming process. 

Somatic cell memory has been reported to be transient and to be attenuated with further passaging in mouse iPSCs [7]. However, these observations remain controversial, as another report detected residual donor-cell type memory in human iPSCs even at late passages [10]. It is intriguing that all the studies reporting a permanent memory of the cell of origin have been conducted in human iPSCs [8,10,14,15]. Thus, the discrepancy between these studies could be due to the different naïve and primed pluripotent cell stages exhibited by mouse and human iPSCs, respectively [16]. We speculate that naïve pluripotent cells might erase the residual donor cell memory more efficiently than primed pluripotent cells, suggesting that the ability to reset the epigenome might correlate with developmental potential. More studies are required to support this hypothesis, which indirectly suggests that somatic cells generated through a direct reprogramming approach might retain a higher level of epigenetic memory than iPSCs. In fact, our data show that the MEF transcriptional signature detected in iNSCs is completely eliminated upon iNdiPSC generation, supporting the hypothesis that reprogramming to pluripotency erases the donor-cell type transcriptional memory more efficiently than reprogramming to a somatic cell state. 

Reprogramming to pluripotency requires several rounds of cell division, which are essential for acquiring the pluripotent cell state and resetting the epigenome [17]. In contrast, direct reprogramming into somatic cells does not involve many cell divisions after viral transduction [18,19]. Furthermore, somatic cells generated via direct reprogramming do not progress through the normal developmental intermediates [5,19]. These mechanistic differences prompt questioning of whether somatic cells differentiated from iPSCs are completely equivalent to their counterparts generated by using a direct reprogramming approach. We have reported the direct reprogramming of fibroblasts into iNSCs that exhibit a gene expression profile similar to brain- and iPSC-derived NSCs [6,20]. iNSCs could differentiate into neurons, astrocytes and oligodendrocytes *in vitro* and *in vivo*, like NSCs established directly from brain or differentiated from iPSCs, [6,20]. Furthermore, iNSCs do not induce tumorigenesis in long-term transplantation assays (Figure S3), as it has also been described for brain- and iPSC-derived NSCs [20,21]. In spite of their similarities, global epigenetic analysis comparing the epigenomes of the NSCs generated by each of the different methods has yet to be performed. As shown for iPSCs, the memory of the cell of origin is more strongly retained at the epigenetic (e.g. methylation patterns) than at the transcriptional level [7,9]. Therefore, epigenetic studies are required to compare directly converted somatic cells and their counterparts differentiated from iPSCs or the corresponding primary cells isolated from *in vivo* tissues. These studies will help to assess whether different reprogramming strategies generate equivalent cell types, which can be equally applied for therapeutic treatment. 

In summary, we have shown for the first time that an induced somatic cell type, namely iNSCs, can be further reprogrammed to pluripotency. The resultant iNdiPSCs proved to be *bona fide* iPSCs. Our results indicate that iNdiPSCs do not maintain the iNSC/NSC transcriptional memory. Furthermore, the residual MEF transcriptional memory, still present in the iNSC population, is lost during the final reprogramming step. In addition, iNdiPSCs do not show a differentiation bias toward either the mesenchymal or neural cell lineage. Therefore, we conclude that reprogramming to pluripotency erases the transcriptional memory of the donor-cell type in a more efficient manner than conversion to a somatic cell state. Further investigations are required to evaluate residual memories from somatic cells generated by direct conversion or by iPSC-differentiation and to assess the impact on future clinical applications. 

## Methods

### Cell Culture Conditions

iNSCs (passage 22) were generated and cultured as previously reported [6]. In addition, MEF 4F iPSCs (passage 7), iNdiPSCs (passage 10), and NSC 2F iPSCs (passage 7) were cultured on feeder cells in 2i medium as described [2]. 4F corresponds to *Oct4*, *Sox2*, *Klf4*, and *Myc*; 2F corresponds to *Oct4* and *Klf4*.

### Replication-Defective Retroviral Production and Transduction

pMX retroviral plasmids coding for mouse *Oct4* (Addgene # 13366) [1] and *Klf4* (Addgene # 13370) [1] were cotransfected with the pCL-ECO packaging plasmid (Addgene # 12371) into 293T cells using Fugene (Roche). Of note, 293T cells were cultured in iNSC medium during the transfection process. Supernatants were collected after 48 h and filtered using 0.45-µm filters (Millipore). iNSCs were plated on gelatin-coated 6-well plates (10^5^ cells/well) one day prior to transduction. 500 µl of each retroviral supernatant plus 6 µg/ml protamine sulfate (Sigma) were used for infection in a final volume of 2.5 ml of iNSC medium. 48 h after transduction, the supernatant containing the viral particles was removed and the transduced cells were further cultured in 2i medium.

### Alkaline phosphatase staining

Cells were washed with phosphate buffered saline (PBS), fixed for 5 min in 4% paraformaldehyde (PFA) at room temperature, washed twice with PBS, and stained with the SIGMA FAST Fast Red TR/Napthol AS-MX kit (Sigma) for 20 min in the dark at RT.

### Immunocytochemistry

Cells were fixed for 5 min in 4% PFA, washed twice in PBS, and permeabilized with 0.1% Triton X-100 for 10 min. After washing twice with PBS, cells were blocked with 5% bovine serum albumin (BSA) (GIBCO) for 1 h while shaking and incubated for 1 h with the primary antibodies diluted in 1% BSA. The cells were then washed twice and incubated for 1 h with the corresponding secondary antibody diluted in 1% BSA. Finally, cells were washed three times and stained with DAPI. All steps were carried out at RT. The antibodies used and their corresponding dilutions are listed in Table S2.

### RNA extraction, cDNA synthesis, and quantitative real time PCR (qRT-PCR)

RNA was extracted using the QIAGEN RNeasy Mini Kit (QIAGEN) and cDNA synthesis was performed using the High-Capacity cDNA Reverse Transcription Kit (Applied Biosystems). qRT-PCR was carried out using iTaq SYBR Green Supermix With ROX (Bio-Rad). The data was plotted using the delta delta Ct algorithm, 2^(-ΔΔCt)^ [22]. Primers are listed in Table S2. 

### Methylation analysis

Methylation analysis was performed as described in our previous publication [6]. Primers are listed in Table S2.

### Chromosome spread

Chromosome spreads were performed as reported previously [2].

### *In vitro* differentiation

Embryoid bodies were generated using the hanging-drop method (600–800 cells per 20 µl drop in MEF medium). Three days later, the embryoid bodies that had formed were transferred onto gelatin-coated dishes and cultured for 10–14 days. Finally, the differentiated embryoid bodies were analyzed for the expression of cell lineage–specific markers by immunostaining or qRT-PCR. MEF medium was composed of DMEM, 20 % fetal bovine serum, 1X MEM non-essential amino acids, 1X L-Glutamine with Pen/Strep, and 0.1 mM β-mercaptoethanol (all GIBCO). 

### Teratoma formation

1 x 10^6^ iNdiPSCs were injected subcutaneously into SCID mice. Tumors were resected after 4 weeks, fixed in 4% PFA, and embedded in paraffin. Paraffin sections were mounted onto glass slides and stained with hematoxylin and eosin. This study was performed in accordance with the recommendations of the Federation of Laboratory Animal Science Associations (FELASA). The corresponding ethics protocol, covering the experiments of teratoma formation in mice, was approved by the Landesamt für Natur, Umwelt und Verbraucherschutz (LANUV) of the state of North Rhine-Westphalia, Germany. Teratoma formation was approved by permit number 87-51-04-2010-A387. In addition, 1 x 10^6^ iNSCs were transplanted into the contused thoracic spinal cord of Sprague-Dawley rats 9 days after injury as previously described [23]. PBS and control NSCs were used as controls. Spinal cords were collected 12 weeks after transplantation, fixed and stained with hematoxylin and eosin to assess the presence of tumors. The Institutional Animal Care and Use Committee of Dankook University approved all animal care and surgical procedures (Approval No. DKU 12-020). Every effort was made to minimize suffering. 

### Microarray analysis

Microarray analysis was performed as described in our previous publication [24]. The data discussed in this publication have been deposited in NCBI’s Gene Expression Omnibus and are accessible through GEO Series accession number GSE44284. Different filter criteria were used in the transcriptional memory analysis. NSC memory in iNdiPSCs: (i) at least 2-fold higher expression in iNdiPSCs compared with ESCs, (ii) at least 2-fold higher expression in iNdiPSCs compared with MEF 4F iPSCs, and (iii) at least 2-fold higher expression in iNSCs compared with MEFs or ESCs. MEF memory in iNdiPSCs: (i) at least 2-fold higher expression in iNdiPSCs compared with ESCs, (ii) at least 2-fold higher expression in iNdiPSCs compared with NSC 2F iPSCs, and (iii) at least 2-fold higher expression in MEFs compared with NSCs or ESCs. MEF memory in MEF 4F iPSCs: (i) at least 2-fold higher expression in MEF 4F iPSCs compared with ESCs, (ii) at least 2-fold higher expression in MEF 4F iPSCs compared with NSC 2F iPSCs, and (iii) at least 2-fold higher expression in MEFs compared with NSCs or ESCs. NSC memory in NSC 2F iPSCs: (i) at least 2-fold higher expression in NSC 2F iPSCS compared with ESCs, (ii) at least 2-fold higher expression in NSC 2F iPSCs compared with MEF 4F iPSCs, and (iii) at least 2-fold higher expression in NSCs compared with MEFs or ESCs. MEF memory in iNSCs: (i) at least 2-fold higher expressed in iNSCs compared with NSCs, and (ii) at least 2-fold higher expressed in MEFs compared with NSCs or ESCs. 

## Supporting Information

Figure S1 **Heat map of potential MEF memory genes in iNSCs.** The filter criteria are listed in the Methods section. Color bar at the top indicates gene expression in log_2_ scale. Red and blue colors represent high and low expression levels, respectively.
(TIF)

Figure S2 **Gene expression levels of selected MEF memory genes were verified in iNSCs using qRT-PCR.** Data were plotted relative to MEFs. Error bars indicate standard error of two different housekeeping genes (*Gapdh* and *Actb*).
(TIF)

Figure S3 **Analysis of tumor formation in rat spinal cords after iNSCs (n=5), control NSCs (cNSCs, n=5) and PBS (vehicle, n=5) transplantation.** Hematoxylin/eosin stained sagittal sections did not show any tumor formation 12 weeks after engraftment.
(TIF)

Table S1 **iNdiPSC reprogramming efficiency.**
(PDF)

Table S2 **List of primers and antibodies used in this study.**
(PDF)
