# Supplementary figures and images for: Reprogramming to Pluripotency through a Somatic Stem Cell Intermediate
Source: PLoS One. 2013 Dec 27;8(12):e85138. doi: 10.1371/journal.pone.0085138 (PMC3874029; doi:10.1371/journal.pone.0085138)

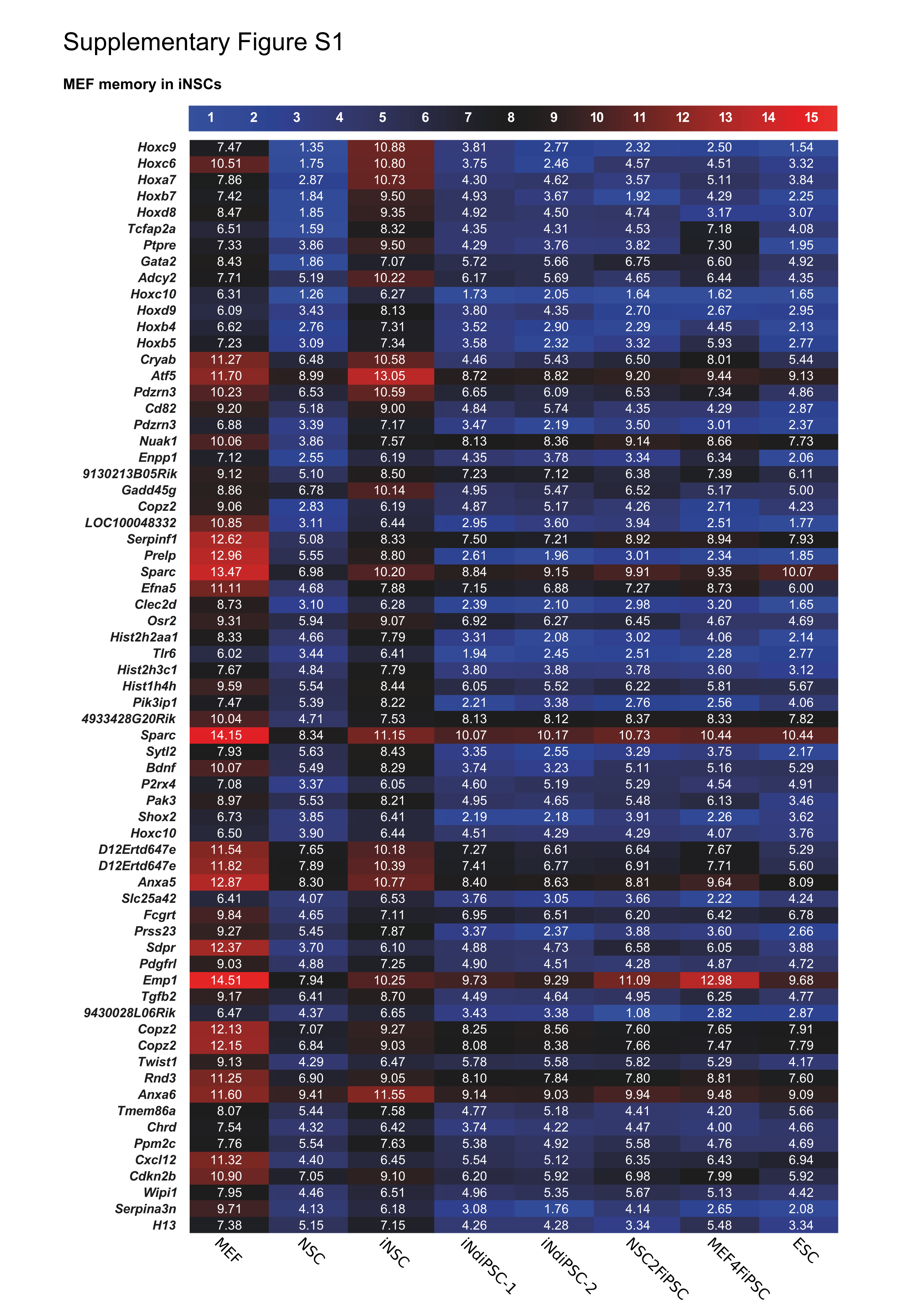

Supplement: Figure S1 — Heat map of potential MEF memory genes in iNSCs. The filter criteria are listed in the Methods section. Color bar at the top indicates gene expression in log2 scale. Red and blue colors represent high and low expression levels, respectively. (TIF) [file pone.0085138.s001.tif]

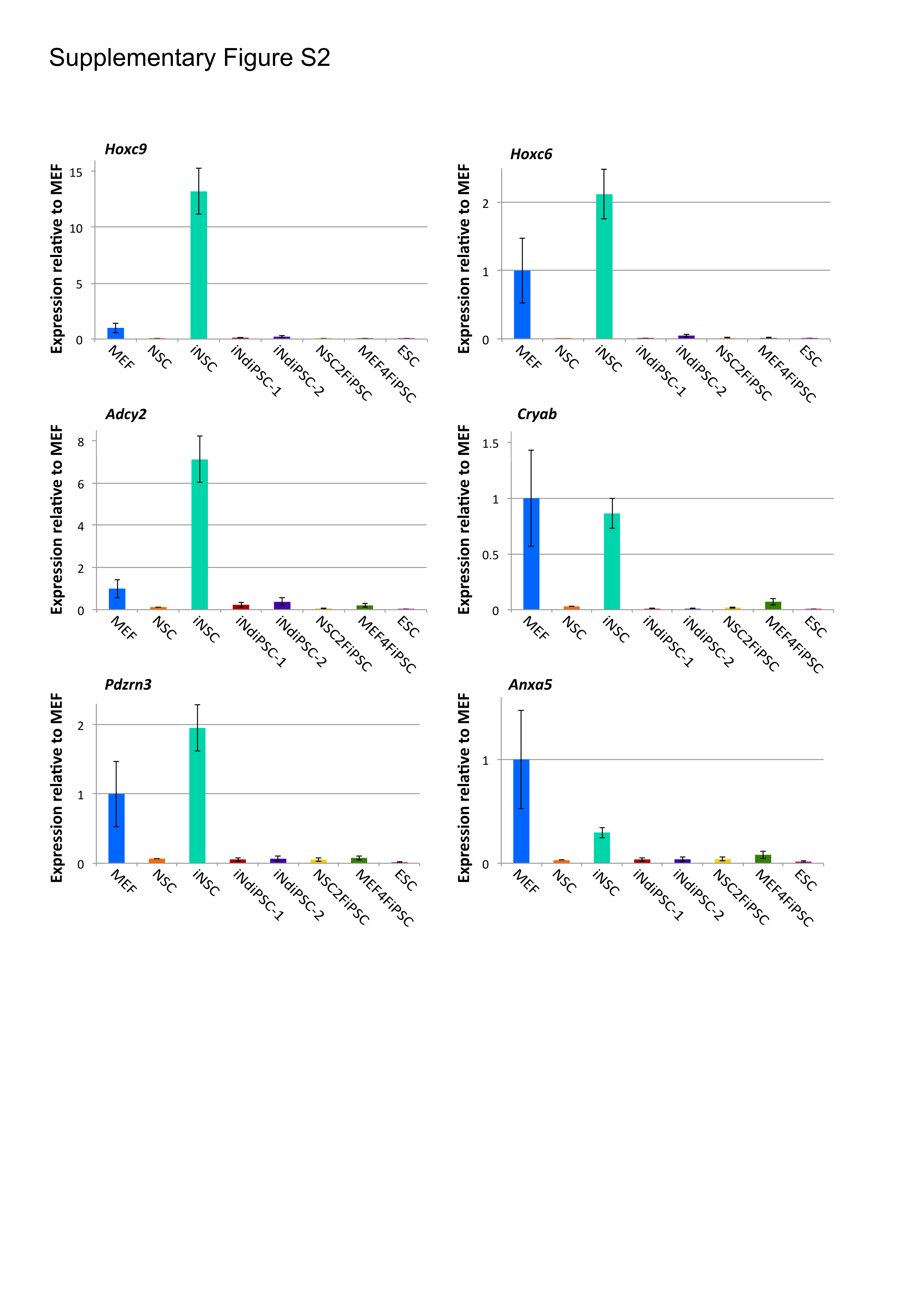

Supplement: Figure S2 — Gene expression levels of selected MEF memory genes were verified in iNSCs using qRT-PCR. Data were plotted relative to MEFs. Error bars indicate standard error of two different housekeeping genes (Gapdh and Actb). (TIF) [file pone.0085138.s002.tif]

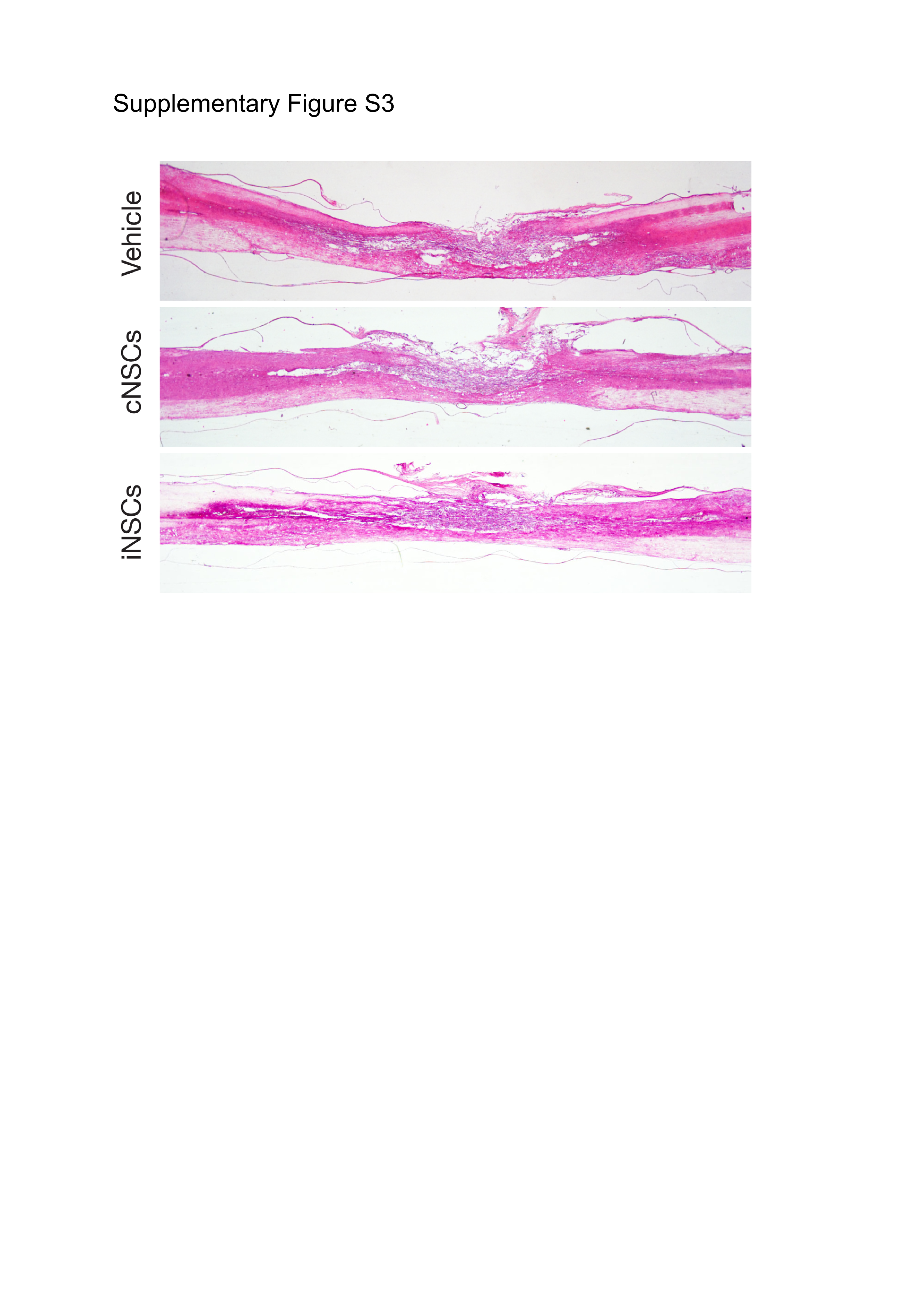

Supplement: Figure S3 — Analysis of tumor formation in rat spinal cords after iNSCs (n=5), control NSCs (cNSCs, n=5) and PBS (vehicle, n=5) transplantation. Hematoxylin/eosin stained sagittal sections did not show any tumor formation 12 weeks after engraftment. (TIF) [file pone.0085138.s003.tif]
