# Supplementary material for: Reprogramming to Pluripotency through a Somatic Stem Cell Intermediate
Source: PLoS One. 2013 Dec 27;8(12):e85138. doi: 10.1371/journal.pone.0085138 (PMC3874029; doi:10.1371/journal.pone.0085138)
Supplement: Table S1 — iNdiPSC reprogramming efficiency. (PDF) [file pone.0085138.s004.pdf]

Supplementary Table S1

	No. of AP+ colonies well #1	No. of AP+ colonies well #2	Mean ± SD	Efficiency
**Experiment 1**	**56**	**44**	**50 ± 8.5**	**0.05%**
**Experiment 2**	**88**	**88**	**88 ± 0**	**0.088%**

Supplementary Table S1

Number of alkaline phosphatase positive (AP+) colonies in two independent reprogramming experiments after 22 and 19 days after transduction, respectively. 100,000 iNSCs were plated in one well of a 6-well plate in duplicates and the reprogramming efficiency was calculated accordingly. SD: standard deviation.
