# Supplementary material for: Reprogramming to Pluripotency through a Somatic Stem Cell Intermediate
Source: PLoS One. 2013 Dec 27;8(12):e85138. doi: 10.1371/journal.pone.0085138 (PMC3874029; doi:10.1371/journal.pone.0085138)
Supplement: Table S2 — List of primers and antibodies used in this study. (PDF) [file pone.0085138.s005.pdf]

Supplementary Table S2

**Primary antibodies**		
Anti-NANOG	REC-RCAB0002P-F, Cosmo Bio Co.	1:1000
Anti-SSEA1	MC-480, Millipore	1:160
Anti-TUBB3	T8660, Sigma	1:2000
Anti-ACTA2	M0851, Dako	1:200
Anti-SOX17	AF1924, R&D Systems	1:100
**Secondary antibodies**		
Goat anti-rabbit	Alexa Fluor 568, A-11011, Invitrogen	1:2000
Rabbit anti-mouse	Alexa Fluor 568, A-11061, Invitrogen	1:2000
Rabbit anti-goat	Alexa Fluor 568, A-11079, Invitrogen	1:2000
**Quantitative real-time PCR primer**		
c-Myc forward	CTGCGTGACCAGATCCCTGA	
c-Myc reverse	GCTTGTGCTCGTCTGCTTGAA	
Klf4 forward	TGTGTCGGAGGAAGAGGAAGC	
Klf4 reverse	ACGACTCACCAAGCACCATCA	
Oct4 forward	TGTTCCCGTCACTGCTCTGG	
Oct4 reverse	TTGCCTTGGCTCACAGCATC	
Sox2 forward	TTCGAGGAAAGGGTTCTTGCTG	
Sox2 reverse	TCCTTCCTTGTTTGTAACGGTCCT	
Nanog forward	GAACGGCCAGCCTTGGAAT	

Nanog reverse	GCAACTGTACGTAAGGCTGCAGAA
Rex1 forward	GGCTGCGAGAAGAGCTTTATTCA
Rex1 reverse	AGCATTTCTTCCCGGCCTTT
Rpl37a forward	ACTTGCTCCTTCTGTGGCAAGAC
Rpl37a reverse	TTCATGCAGGAACCACAGTGC
pMX-*Oct4* forward	GATCCCAGTGTGGTGGTACGG
pMX-*Oct4* reverse	GGCGAAGTCTGAAGCCAGGT
pMX-*Sox2* forward	GATCCCAGTGTGGTGGTACGG
pMX-*Sox2* reverse	GGCTTCAGCTCCGTCTCCAT
pMX-*Brn4* forward	GGGTGGACCATCCTCTAGACT
pMX-*Brn4* reverse	ATGGACAAGGGAGCTGGAAC
pMX-*Myc* forward	GATCCCAGTGTGGTGGTACGG
pMX-*Myc* reverse	TCGAGGTCATAGTTCCTGTTGGTG
pMX-*Klf4* forward	GATCCCAGTGTGGTGGTACGG
pMX-*Klf4* reverse	GTGGAGAAGGACGGGAGCAG
Nestin forward	GCTGGACTGGAACTCGAGC
Nestin reverse	GATGGGTGAATGGCCTCCTC
Tpmt forward	GCTTGCTGTCTTCAGTTGCTTCA
Tpmt reverse	AGAAGGCCTGGCGTTTCAGA
Acsl6 forward	GGCTAAGAGACCGGAGCTGAGA
Acsl6 reverse	TGGAGCTTCACACGGAGACTAAGTAA
Atp1b2 forward	TACTCAGGCGGGCTTGACCT
Atp1b2 reverse	CCCTGGGAAGGAGAATTTGAAAG
Hebp2 forward	CCTACGCTACATGACAGGTGTGC

Hebp2 reverse	GTTGGACAGAGAATGTCCATACAACCT
Ly6a forward	CCCAATGACCTCCACCCTTG
Ly6a reverse	CACTACTCCCACCTTGGAGCTTCT
Crct1 forward	TCACCCTGTTCTTAGACCTGTCCTC
Crct1 reverse	TGCACAACCTCCTGCCCATA
Ly6c1 forward	GGACTGCAGTGCTACGAGTGCTA
Ly6c1 reverse	GCAATGCAGAATCCATCAGAGG
Cxcl1 forward	AGGACATGTGTGGGAGGCTGT
Cxcl1 reverse	AAATGTCCAAGGGAAGCGTCA
Hoxc9 forward	TTGCGATGTGGGAGGGTTAAG
Hoxc9 reverse	CAGTTTCTCTCCTGCCTCCTCCT
Hoxc6 forward	AAATGCCCAGTCCAGGCAAA
Hoxc6 reverse	GAAATATTCACACAGAAACGGTCACAG
Adcy2 forward	GTCTCATTGCCAGTGGTCATCC
Adcy2 reverse	ACGTGCTGCTGCCACAAGA
Cryab forward	GAGTCCTCACTGTGAATGGACCAA
Cryab reverse	GGCGACAGCAGGCTTCTCTT
Pdzrn3 forward	TGGACGCTTAAAGCATGTTTGC
Pdzrn3 reverse	ACAGGTCTCCATCCGCGACT
Anxa5 forward	GCCCACCATCAGCTTTCCTC
Anxa5 reverse	AAGGGCGGGACACTGCTTT
Gapdh forward	CCAATGTGTCCGTCGTGGAT
Gapdh reverse	TGCCTGCTTCACCACCTTCT
Actb forward	ACTGCCGCATCCTCTTCCTC

Actb reverse	CCGCTCGTTGCCAATAGTGA
Sox17 forward	AGCCATTTCCTCCGTGGTGT
Sox17 reverse	AACACTGCTTCTGGCCCTCAG
T forward	TTGAACTTTCCTCCATGTGCTGA
T reverse	TCCCAAGAGCCTGCCACTTT
Bmp4 forward	GGCTGGCCATTGAGGTGACT
Bmp4 reverse	TCGGCTGATTCTGACATGCTG
Sox1 forward	GGCCGAGTGGAAGGTCATGT
Sox1 reverse	TCCGGGTGTTCCTTCATGTG
Otx2 forward	TGTCCCAGGCTCATTCACGTTTCA
Otx2 reverse	TCGCACAATCCACACAGCCCT
Pax6 forward	CGCGGATCTGTGTTGCTCAT
Pax6 reverse	CTTAAATCCATGGCAAATCTTGTCG
Dppa3 forward	GCCGCACAGCAGATGTGAA
Dppa3 reverse	AAATCTGGATCGTTGTGCATCCT
Bglap forward	TCTCTGACCTCACAGATGCCAAG
Bglap reverse	AGCGCCGGAGTCTGTTCACT
Sp7 forward	CCTATGCTCCGACCTCCTCAAC
Sp7 reverse	GATGTGAGGCCAGATGGAAGC
Runx2 forward	CACCAAGTAGACGCAGATGGTCA
Runx2 reverse	CATGGACCGTGGTGTGCTTC
Tubb3 forward	TGATGACGAGGAATCGGAAGC
Tubb3 reverse	GGACAGATGCTGCTTGTCTTGG
Map2 forward	TCAGGCATTTCTCCAAGATTGATG

Map2 reverse	CCTACAGAGGGACTTGGCCTCA	
Ibsp forward	GGCTATTGATCAAGCAGCACACA	
Ibsp reverse	TGCGCAGTTAGCAATAGCACAAA	
**Bisulfite methylation analysis primer**		
Oct4 promoter 1st forward	TTTGTTTTTTATTTATTTAGGGGG	299 bp, 45 °C
Oct4 promoter 1st reverse	ATCCCCAATACCTCTAAACCTAATC	299 bp, 45 °C
Oct4 promoter 2nd forward	GGGTTAGAGGTTAAGGTTAGAGGG	161 bp, 45 °C
Oct4 promoter 2nd reverse	CCCCCACCTAATAAAAATAAAAAAA	161 bp, 45 °C
Nestin enhancer 1st forward	TAAAGAGGTTGTTTGGTTTGGTAGT	394 bp, 45 °C
Nestin enhancer 1st reverse	CTATTCCACTCAACCTTCCTAAAA	394 bp, 45 °C
Nestin enhancer 2nd forward	TAGTTTTTAGGGAGGAGATTAGAGG	188 bp, 55 °C
Nestin enhancer 2nd reverse	CTCTTACCCCAAACACAACTAAAAC	188 bp, 55 °C

Supplementary Table S2

List of primary and secondary antibodies, as well as primers used for quantitative real-time PCR and bisulfite methylation analysis.
